# Ultrastructural Changes and Death of* Leishmania infantum* Promastigotes Induced by* Morinda citrifolia* Linn. Fruit (Noni) Juice Treatment

**DOI:** 10.1155/2016/5063540

**Published:** 2016-05-22

**Authors:** Fernando Almeida-Souza, Noemi Nosomi Taniwaki, Ana Cláudia Fernandes Amaral, Celeste da Silva Freitas de Souza, Kátia da Silva Calabrese, Ana Lúcia Abreu-Silva

**Affiliations:** ^1^Laboratório de Imunomodulação e Protozoologia, Instituto Oswaldo Cruz, Fiocruz, 21040-900 Rio de Janeiro, RJ, Brazil; ^2^Departamento de Patologia, Universidade Estadual do Maranhão, 65055-310 São Luís, MA, Brazil; ^3^Unidade de Microscopia Eletrônica, Instituto Adolf Lutz, 01246-000 São Paulo, SP, Brazil; ^4^Laboratório de Plantas Medicinais e Derivados, Farmanguinhos, Fiocruz, 21041-250 Rio de Janeiro, RJ, Brazil

## Abstract

The search for new treatments against leishmaniasis has increased due to high frequency of drug resistance registered in endemics areas, side effects, and complications caused by coinfection with HIV.* Morinda citrifolia* Linn., commonly known as Noni, has a rich chemical composition and various therapeutic effects have been described in the literature. Studies have shown the leishmanicidal activity of* M. citrifolia*; however, its action on the parasite has not yet been elucidated. In this work, we analyzed leishmanicidal activity and ultrastructural changes in* Leishmania infantum* promastigotes caused by* M. citrifolia* fruit juice treatment.* M. citrifolia* fruit extract showed a yield of 6.31% and high performance liquid chromatography identified phenolic and aromatic compounds as the major constituents. IC_50_ values were 260.5 *µ*g/mL for promastigotes and 201.3 *µ*g/mL for intracellular amastigotes of* L. infantum* treated with* M. citrifolia*. Cytotoxicity assay with J774.G8 macrophages showed that* M. citrifolia* fruit juice was not toxic up to 2 mg/mL. Transmission electron microscopy showed cytoplasmic vacuolization, lipid inclusion, increased exocytosis activity, and autophagosome-like vesicles in* L. infantum* promastigotes treated with* M. citrifolia* fruit juice.* M. citrifolia* fruit juice was active against* L. infantum* in the* in vitro* model used here causing ultrastructural changes and has a future potential for treatment against leishmaniasis.

## 1. Introduction

Due to the continental dimensions of Brazil there are various parts of its territory with difficult access. Consequently, there is a limit to public health resources and a tendency for the inhabitants of these remote regions not to get the necessary government health benefits. This geographical isolation contributes to strengthening the local traditional medical practices and other natural resources to treat diseases, including parasitic diseases such as leishmaniasis [1].

Leishmaniasis is caused by protozoan parasites transmitted through the bites of infected female sandflies (usually* Phlebotomus* or* Lutzomyia*). The disease appears in three clinical forms: the visceral form, also known as kala-azar, is usually fatal within 2 years if left untreated; the cutaneous form, causing skin ulcers; and the mucocutaneous form, which invades the mucous membranes of the upper respiratory tract, causing gross mutilation by destroying soft tissues in the nose, mouth, and throat [2]. The disease, which is prevalent in 98 countries and 3 territories on 5 continents, has approximately 1.3 million new cases annually, of which 300,000 are visceral and 1 million are cutaneous or mucocutaneous. These numbers show the importance of this disease in public health, including Brazil [3].

Treatment for leishmaniasis was first introduced by Vianna in 1912. Organic compounds of antimony are the drugs of choice in treating this disease, and amphotericin B was introduced recently. Both treatments present several side effects, are highly toxicity, and have an elevated cost which has led to the search for new alternatives. The search for a leishmanicidal agent of low toxicity and high efficiency is a challenge and has involved several research groups around the world [4]. Herbal remedies have gained a lot of attention in this area as a potential source to obtain new compounds with therapeutic activities.

*Morinda citrifolia* Linn. is a small plant native to Southeast Asia, commonly known as Noni, and one of the most significant sources of traditional medicine in those countries. Due to the various ethnopharmacological activities associated with this plant, it is now cultivated all over the world, including Brazil. Studies have shown the efficacy of Noni in the treatment of pain and inflammatory reactions [5] and antitumoral activity [6]. Activity against bacteria [7] and fungi [8] has also been observed. Recently, the* in vitro* activity of morindicone and morinthone isolated from the stem of* M. citrifolia* was described against* L. major*. Moreover, a clinical study was carried out to determine the efficiency of a topical ointment with* M. citrifolia* stem extract against cutaneous leishmaniasis, and there was an excellent response in 50% and a good improvement in 30% of the 40 patients evaluated [9]. Therefore, to demonstrate the action of* M. citrifolia* against promastigotes of* Leishmania* and evaluate the ultrastructural changes caused by such treatment, this study evaluated promastigotes forms of* Leishmania infantum* treated with* M. citrifolia* fruit juice by electron microscopy.

## 2. Materials and Methods

### 2.1. Plant Material

*M. citrifolia* fruits were collected in November 2011 from São Luís (S2°31 W44°16), Maranhão, in the Brazilian Legal Amazon at 24 m above sea level. Fruits were collected when the exocarp was translucent. The plant material was identified by Ana Maria Maciel Leite, and the voucher specimen number 2000346 was deposited at the Herbário Professora Rosa Mochel, Universidade Estadual do Maranhão. In the laboratory, the fruits were washed with distilled and sterilized water, dried at 25°C, and placed in sterile glass bottles for 3 days to drain off the extract released. This liquid was centrifuged twice at 4000 rpm for 15 minutes; the supernatant was lyophilized and stored at −20°C [8]. The lyophilized* M. citrifolia* fruit juice was dissolved in DMSO and dilutions with different concentrations in culture medium were made immediately before use. The concentration of DMSO in medium did not exceed 1%.

### 2.2. High Performance Liquid Chromatography Coupled with Diode Array and Evaporative Light Scattering Detectors (HPLC-DAD-ELSD)

The HPLC chromatographic profile of the* M. citrifolia* fruit juice was performed on a Shimadzu LC-10Avp equipped with two LC-8Avp pumps, controlled by a CBM-10A interface module, an automatic injector with two detectors, a diode array detector SPD-M10A (DAD), and an evaporative light scattering detector (ELSD) with a drift tube temperature setting of 40°C, using nitrogen as the nebulizer gas and gain at 4.0. HPLC grade solvents and Milli-Q water were used and the analysis was performed on a reversed phase LiChrospher C18 column (4.6 mm × 250 mm; 5 *μ*m, Waters). The mobile phase was water (A) and methanol (B), with the following gradient composition: (0–20 min) 5–20% (B), (20–30 min) 20%–35% (B), and (30–35 min) 35% (B). The UV chromatogram was obtained at 365 nm. The sample injection volume was 10 *µ*L. A constant flow of 1 mL/min was used during the analysis. Before analysis 5.0 mg of the extract was dissolved in 1.0 mL of Milli-Q water and the mixture was centrifuged.

### 2.3. Parasites

Promastigote forms of* L. infantum* (MCAN/BR/2008/1112) were cultured at 26°C in Schneider's Insect medium (Sigma-USA) supplemented with 10% fetal bovine sera (Gibco-USA), 100 U/mL of penicillin (Gibco-USA), and 100 *µ*g/mL of streptomycin. The cultures used had a maximum of ten* in vitro* passages.

### 2.4. Animals

Female BALB/c mice of 4–6 years old were purchased from Centro de Criação de Animais de Laboratório do Instituto Oswaldo Cruz, Rio de Janeiro, and maintained under pathogen-free conditions. The animals were handled in accordance with Guidelines for Animal Experimentation of the Colégio Brasileiro de Experimentação Animal. The local Ethics Committee on Animal Care and Utilization approved all procedures involving the animals (CEUA FIOCRUZ-LW72/12).

### 2.5. Cells Culture

The macrophage J774.G8 line was cultured in RPMI 1640 medium (Sigma, USA) supplemented with 10% fetal bovine sera, penicillin (100 U/mL), and (100 *µ*g/mL) streptomycin, at 37°C and 5% CO_2_. Female BALB/c mice were inoculated with 3 mL of sodium thioglycolate 3% and after 72 hours peritoneal macrophages were harvested with PBS solution. The harvest was centrifuged at 4000 rpm and the cells suspended in RPMI medium supplemented as described before and cultured at 37°C and 5% CO_2_.

### 2.6. Activity against Promastigote Forms

Promastigote forms of* L. infantum* (10^6^ parasites/mL) from a 2–4-day-old culture were placed in 96-well plates in the presence of different concentrations of* M. citrifolia* fruit juice (960–30 *µ*g/mL), in a final volume of 200 *µ*L per well, for 72 hours. Wells without parasites were used as blank and wells with only parasites were used as control. After the treatment, the viability of parasites was evaluated by the tetrazolium-dye (MTT) colorimetric modified method [10]. MTT (5 mg/mL), a volume equal to 10% of the total, was added to each well. After 2 hours, the plate was centrifuged at 4000 rpm; then the supernatant was removed from each well and 100 *µ*L of DMSO was added to dissolve the formazan. The absorbance was analyzed on a spectrophotometer at a wavelength of 540 nm. The data was normalized according to the formula

(1) %  survival=DO  sample−DO  blankDO  control−DO  blank×100.

The results were used to calculate IC_50_ (50% inhibition of parasite growth). Amphotericin B was used as the reference drug.

### 2.7. Activity against Intracellular Amastigotes

Peritoneal macrophages were cultured in 24-well plates (10^5^ cells/well), with coverslips, at 37°C and 5% CO_2_. The cells were infected with promastigote forms of* L. infantum* using a ratio of 10 : 1 parasite/cell, and after 2 hours the cells were washed three times with PBS to remove free parasites. The infected cells were treated with different concentrations of* M. citrifolia* fruit juice (480–30 *μ*g/mL) in triplicate for 24 hours. The coverslips with the infected and treated cells were fixed with Bouin, stained with Giemsa, and examined by light microscopy. The inhibition percentage was calculated and IC_50_ was calculated with the GraphPad Prism software. Amphotericin was used as the reference drug.

### 2.8. Cytotoxicity Assay

J774.G8 macrophages were cultured in 96-well plates (5 × 10^5^ cells/mL) with different concentrations of* M. citrifolia* fruit juice (2000–1.8 *µ*g/mL) to a final volume of 200 *µ*L per well, at 37°C and 5% CO_2_. Wells without cells were used as blank and wells with only cells were used as control. After 24 hours, the cells were fixed with 10% trichloroacetic acid for 1 hour at 4°C, stained with Sulforhodamine B (Sigma, USA) solution 0.4% in 1% acetic acid for 30 minutes, and washed with 1% acetic acid solution. Sulforhodamine B was solubilized in 200 *µ*L of 10 mM tris-base solution and the plate was read in a spectrophotometer at 540 nm wavelength [11]. The data was normalized following the formula described earlier. The results were used to calculate the cell cytotoxicity by 50% (CC_50_) with the GraphPad Prism 5.

### 2.9. Transmission Electron Microscopy

Promastigote forms of* L. infantum* were treated with* M. citrifolia* fruit juice at concentrations of 480, 240, 120, 60, and 30 *µ*g/mL for 24 hours. The parasites were fixed with 2.5% glutaraldehyde (Sigma, USA) in 0.1 M sodium-cacodylate buffer, pH 7.2 overnight. Parasites were washed three times with 0.1 M sodium-cacodylate buffer and postfixed in a solution containing 1% osmium tetroxide, 0.8% ferrocyanide, and 5 mM calcium chloride, washed in 0.1 M sodium-cacodylate buffer, dehydrated in graded acetone, and embedded in epoxy resin. Ultrathin sections were stained with uranyl acetate and lead citrate and examined in a transmission electron microscope JEM-1011 (JEOL, Japan).

### 2.10. Statistical Analysis

The values were expressed as mean ± SD. The results were analyzed statistically by Analysis of Variance (ANOVA) followed by the Tukey test. The analyses were performed with the software GraphPad Prism 5.0.4. Differences were considered significant when *p* < 0.05.

## 3. Results and Discussion

**Figure 1:**
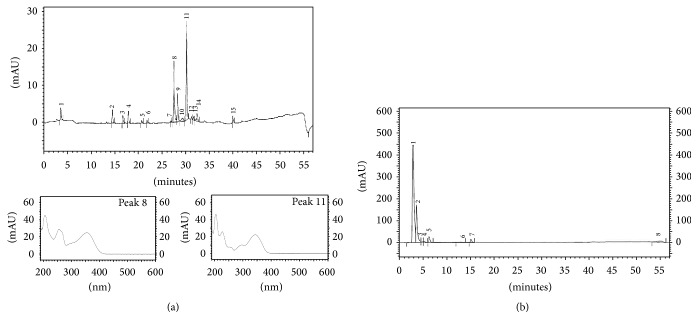
High Performance Liquid Chromatography coupled with diode array detector (a) and evaporative light scattering detector (b) of* Morinda citrifolia* fruit juice at 365 nm. (a) Peaks 8 and 11: highlight of the UV spectra.

The* M. citrifolia* fruit juice was brown, translucent, and of medium viscosity, with its characteristic odor and pH 3.94. After lyophilization, the juice yielded 6.31% of a highly hygroscopic powder. The constituents of* M. citrifolia* fruit juice were analyzed by HPLC-DAD-ELSD and the analysis of the chromatograms obtained from both detectors showed peaks related to compounds with sensitivity in the UV region. The LC-DAD and LC-ELSD chromatograms are presented in Figure 1. The major peaks of the UV-365 nm chromatogram were associated with the characteristic UV spectra of flavonoid (peak 8) and anthraquinones (peak 11). The ELSD fingerprint showed an intense peak at 3.2 min which was not observed in the DAD chromatogram. This signal could be related to the polysaccharides previously reported in* M. citrifolia* that have significant antitumoral activity [12]. Polysaccharides from* Echinacea purpurea* showed activity against* Leishmania enriettii* [13] and the presence of these substances in the phytochemical fingerprint showed the probable chemical potential of the fruit extract against protozoa such as the* Leishmania* genus. This potential was demonstrated through* in vitro* leishmanicidal activity of the* M. citrifolia* fruit juice against* L. infantum*.

**Figure 2:**
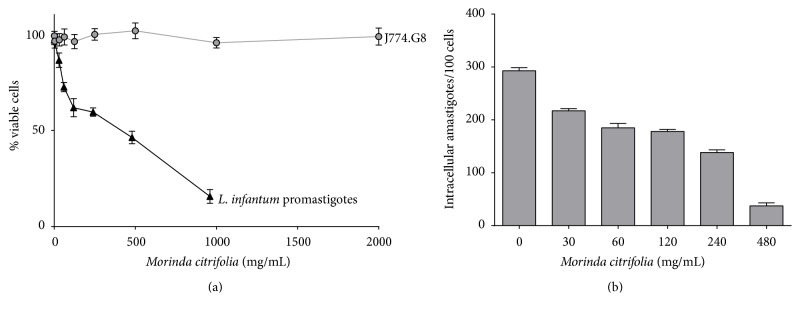
Leishmanicidal activity and* in vitro* cytotoxicity of* Morinda citrifolia* fruit juice. (a) Viability of* Leishmania infantum* promastigotes and J774.G8 macrophages treated with* M. citrifolia* for 72 and 24 hours, respectively. (b) Intracellular amastigotes in BALB/c peritoneal macrophages treated for 24 hours. Data represent mean ± SD of at least three independent experiments realized in quintuplicate.

The effect of* M. citrifolia* fruit juice on the promastigote forms of* L. infantum* was monitored for 72 hours.* M. citrifolia* fruit juice produced a dose-dependent reduction in the proliferation of the parasite (Figure 2(a)), with growth inhibition of 50% of the promastigotes at a concentration of 260.5 *µ*g/mL (Table 1). The values are considered promising when compared with other fruit extracts. The crude extract from the fruit* Momordica charantia* showed an IC_50_ under 600 *µ*g/mL for* L. donovani* promastigotes [14].

There are few data about* in vitro* leishmanicidal activity of* M. citrifolia* constituents in the literature. A clinical trial on the antileishmanial activity of* M. citrifolia* showed good activity for two anthraquinones isolated from stem extract, morindicone and morinthone [9]. Anthraquinones also have been isolated from* M. lucida*, a plant of same gender of* M. citrifolia,* and presented activity against the growth of* Plasmodium falciparum* and promastigotes of* L. major in vitro* [15].

Trying to find compounds responsible for* in vitro* leishmanicidal activity, the* M. citrifolia* fruit juice was submitted to a column partition and, interestingly, the partitions showed IC_50_ values above the value obtained for the full juice (data not shown). This result indicates that various substances present in the* M. citrifolia* fruit juice contribute to the leishmanicidal activity, probably, synergistically, corroborating previous studies where more than one molecule presented biological activity [16, 17].

Although the leishmanicidal activity* in vitro* against promastigotes is used by many researchers as a screening test to search for new drugs for the treatment of leishmaniasis, the positive result of this test alone cannot be considered as an indicator of potential drug action. Activity against intracellular amastigotes is necessary and is perhaps the most effective way to relate the* in vitro* activity of a substance with its possible effectiveness* in vivo*. Thus we also evaluated* M. citrifolia* activity against intracellular amastigotes (Figure 2(b)).

**Figure 3:**
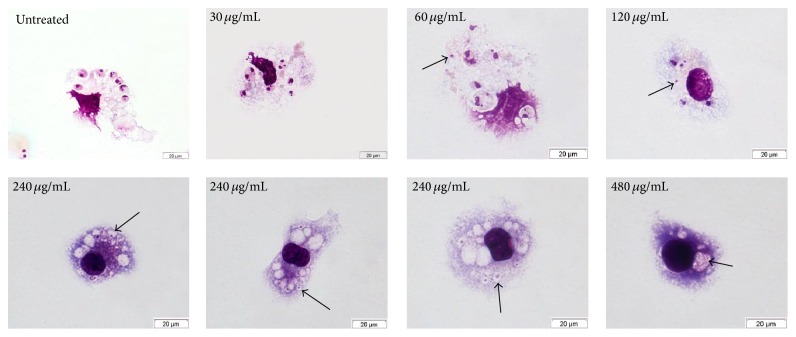
Light microscopy of BALB/c peritoneal macrophages infected with* Leishmania amazonensis* and treated with* Morinda citrifolia* fruit juice. Arrows indicate vacuoles with probable remains of intracellular amastigotes.

As shown in Table 1, there is an increase in activity of the* M. citrifolia* fruit juice against intracellular amastigotes compared with the activity against promastigotes. The IC_50_ value decreased for intracellular amastigotes with a value of 201.3 *µ*g/mL. When observed by light microscopy, macrophages showed vacuoles with probable remains of intracellular amastigotes (Figure 3). This result indicates the possible action of the* M. citrifolia* fruit juice on macrophage activation and modulation, as already shown in previous works, such as the decreased production of IL-4 and increased production of TNF-*α*, IL-1*β* [12], INF-*γ*, and NO [18].

To ensure that* M. citrifolia* fruit juice was only acting on intracellular amastigotes, without causing damage to the host cell, the cytotoxicity in J774.G8 lineage macrophages was investigated by the Sulforhodamine B method (Figure 2(a)). This colorimetric method is based on the quantification of total protein by binding anionic Sulforhodamine B electrostatic crystals with cellular proteins. No cytotoxicity was observed at the concentrations analyzed and the selectivity index for* M. citrifolia* fruit juice was at least 3.3-fold higher than amphotericin B (Table 1).

Macrophages were used to assess the toxicity* in vitro* for being target cells of infection by* Leishmania*. The analysis of cytotoxicity against macrophages J774.G8 shows the low cytotoxicity of* M. citrifolia* fruit juice. This data becomes more relevant when analyzed together with IC_50_ to intracellular amastigote forms, generating SI higher than 9.9 that falls within the generic hit selection criteria of SI to new compounds for infectious diseases from Japanese Global Health Innovative Technology [19]. Indeed, as the generic hit criteria must be applied to phytotherapy with some reservations, the selective index is the most reliable criterion to assess the safety of extracts, essential oils, or others natural products. Besides, the leishmanicidal activity of* M. citrifolia* must be analyzed in addition to immunomodulatory effects and toxicity in posterior studies.

**Figure 4:**
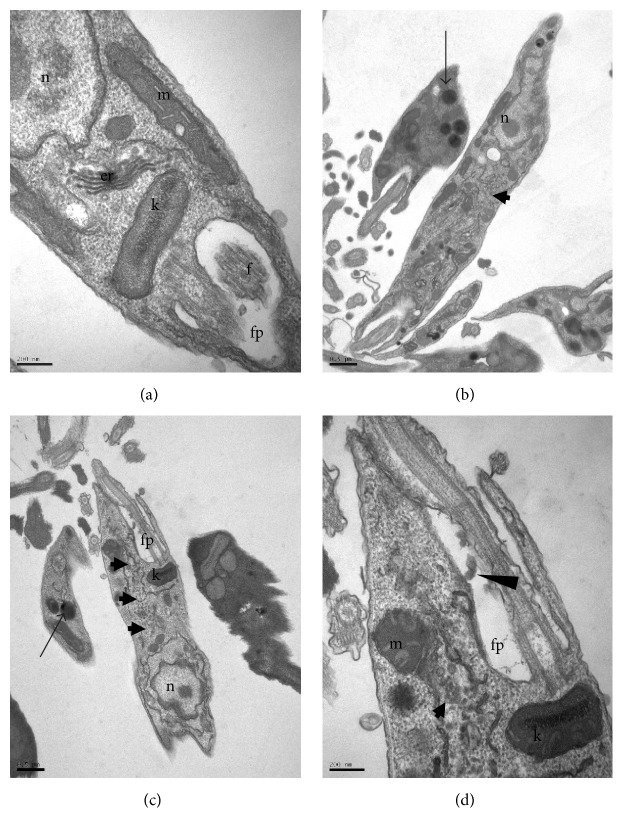
Ultrastructure of* Leishmania infantum* promastigotes incubated for 24 hours, at 26°C, with* Morinda citrifolia* fruit juice. (a) Control. (b–d) Promastigote treated with 30 *µ*g/mL. (b-c) Electron-dense vesicles (arrows) and granular material throughout the cytoplasm (block arrows). (d) Membranes in flagellar pocket (arrowhead). k: kinetoplast, m: mitochondria, n: nucleus, pf: flagellar pocket, f: flagellum, and er: endoplasmic reticulum.

**Figure 5:**
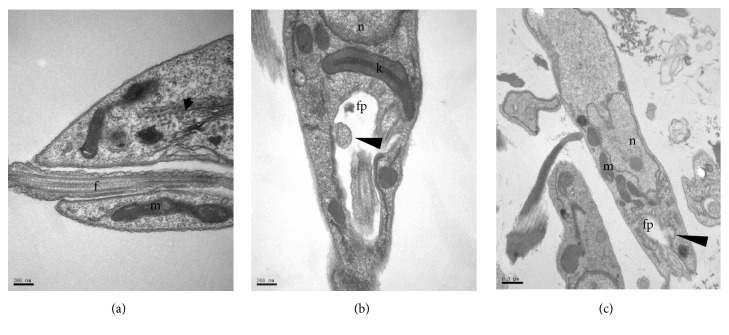
Ultrastructure of* Leishmania infantum* promastigotes incubated for 24 hours, at 26°C, with* Morinda citrifolia* fruit juice. (a–c) Promastigote treated with 60 *µ*g/mL. Electron-dense vesicles (arrows) and granular material throughout the cytoplasm (block arrows). Vesicles breaking up in flagellar pocket (arrowhead). k: kinetoplast, m: mitochondria, n: nucleus, pf: flagellar pocket, and f: flagellum.

**Figure 6:**
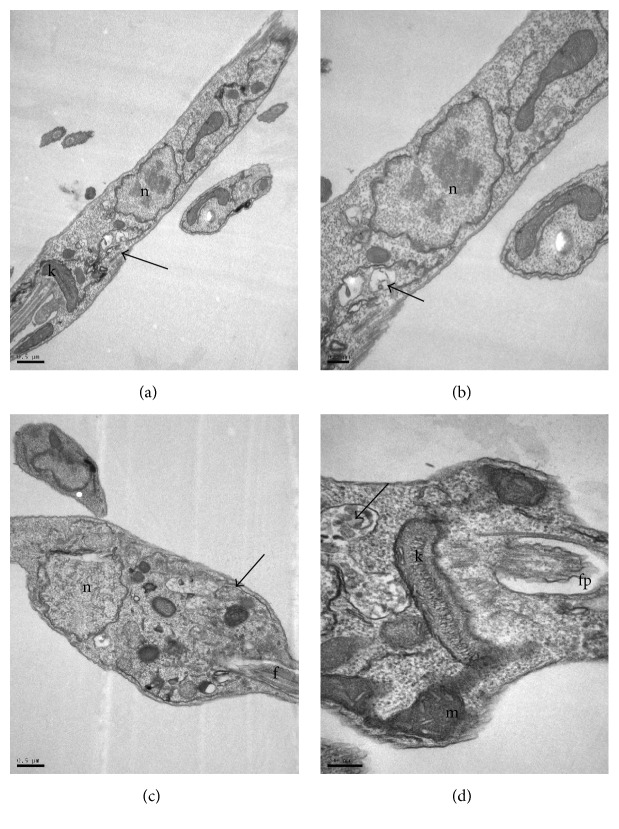
Ultrastructure of* Leishmania infantum* promastigotes incubated for 24 hours, at 26°C, with* Morinda citrifolia* fruit juice. (a-b) Promastigotes treated with 120 *µ*g/mL. (c-d) Promastigotes treated with 240 *µ*g/mL. Vesicles with granular material throughout the cytoplasm (arrows). Vesicles with autophagosome-like material (asterisks). k: kinetoplast, m: mitochondria, n: nucleus, pf: flagellar pocket, and f: flagellum.

**Figure 7:**
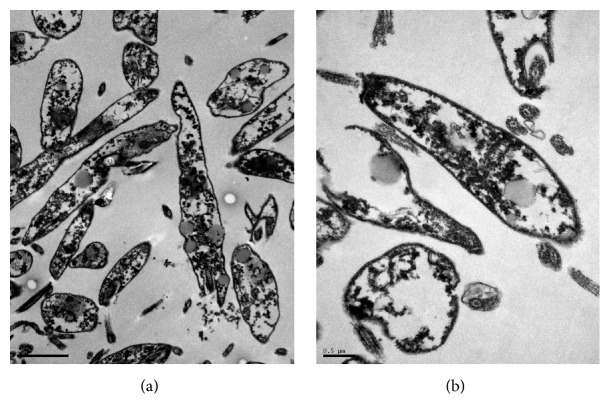
Ultrastructure of* Leishmania infantum* promastigotes incubated for 24 hours, at 26°C, with 480 *µ*g/mL of* Morinda citrifolia* fruit juice. (a-b) Promastigotes with loss of membrane integrity.

The transmission electron microscopy analysis of* L. infantum* promastigotes treated with the* M. citrifolia* fruit juice was performed to determine the ultrastructural changes. Photomicrographs of promastigotes (Figures 4, 5, 6, and 7) showed the degree of damage after 24 hours of treatment. The parasites without treatment showed normal morphology (Figure 4(a)).

The observation of* L. infantum* promastigotes treated with 30 *µ*g/mL of juice showed vacuolization of the cytoplasm, some with electron-dense regions inside, and this became more evident at higher concentrations (Figures 4(b) and 4(c)). Similar structural changes have also been described in* L. amazonensis* treated with essential oils [20]. In these, the vacuoles are associated with entry of substances by simple diffusion, caused by increased permeability of the membrane due to the compounds in the essential oils.

Vesicles in the flagellar pocket were observed in promastigotes treated with 30 and 60 *µ*g/mL of* M. citrifolia* fruit juice (Figures 4(d), 5(b), and 5(c)). The presence of vesicles in flagellar pockets indicates an intense exocytic activity in the region of the flagellar pocket. These changes have also been reported in promastigotes of* L. amazonensis* treated with inhibitors of ergosterol synthesis, such as 22,26-azasterol [21]. The increased activity in the region of the exocytic flagellar pocket may be the result of an abnormal secretion of lipids, which accumulate as a consequence of drug action or indicate an exacerbated production of proteins by cells in an attempt to survive [22].

Membrane structures in the cytoplasm were observed in treatments with 30, 60, 120, and 240 *µ*g/mL of* M. citrifolia* fruit juice (Figures 4(b)–4(d), 5(a), 6(a) and 6(b)). These structures are membranes of the endoplasmic reticulum dispersed in the cytoplasm and are probably involved in the recycling of abnormal organelles. The presence of autophagosome-like vesicle material in promastigotes treated with 240 *µ*g/mL of* M. citrifolia* fruit juice shows an autophagic process (Figures 6(c) and 6(d)), suggesting a remodeling of organelles irreversibly damaged by the treatment. Autophagy can serve as a protective mechanism by recycling macromolecules and removing damaged organelles, but excessive autophagy can result in cell death [23]. To try to survive the effects caused by* M. citrifolia* fruit juice treatment, parasites may react triggering autophagic events, and this exacerbated autophagic response could lead to death, as observed in parasites treated with the 480 *µ*g/mL of juice.

The treatment with 480 *µ*g/mL of* M. citrifolia* fruit juice for 24 hours induced severe cellular damage (Figures 7(a) and 7(b)), with the extravasation of cytoplasmic contents and loss of cellular integrity. The progression of ultrastructural changes is related to increasing drug concentrations, reaching its apex with parasite destruction at higher drug concentrations, and showing the direct action of* M. citrifolia* fruit juice on the parasite and its dose-dependent action. No changes were observed in the nucleus, the mitochondria, the flagellum, the kinetoplast, or the subpellicular microtubules.

## 4. Conclusion

*M. citrifolia* fruit juice showed leishmanicidal activity against* L. infantum* promastigote, causing ultrastructural changes such as cytoplasmic vacuolization, lipid inclusion, increased exocytosis activity, autophagosome-like vesicles, loss of cellular integrity, and death of the parasite. Considering the activity and the alterations observed against promastigote forms of* L. infantum*, further studies must be conducted to evaluate the potential of* M. citrifolia* fruit juice in leishmaniasis treatment.

## Figures and Tables

**Table 1:** Activity against promastigotes and intracellular amastigotes of *Leishmania infantum*, cytotoxicity in peritoneal macrophages from BALB/c and selectivity index of *Morinda citrifolia* fruit extract treatment and amphotericin B.

Compounds	IC_50_ (*µ*g/mL)		CC_50_	SI
	Promastigote	Intracellular amastigote	J774.G8	
*Morinda citrifolia* fruit juice	260.5 ± 0.044	201.3 ± 0.175	>2000	>9.9
Amphotericin B	3.1 ± 0.230	0.9 ± 0.121	2.7 ± 0.156	3.0

IC_50_: inhibitory concentration of 50% parasites. CC_50_: cytotoxicity concentration of 50% cells. SI: selectivity index. Data are presented as the mean ± SD of three independent experiments realized at least in triplicate.
